# Investigations on Green Blends Comprising Biodegradable Polymer and Ionic Liquid

**DOI:** 10.3390/polym8120444

**Published:** 2016-12-21

**Authors:** Li-Ting Lee, Chun-Ting Yang

**Affiliations:** Department of Materials Science and Engineering, Feng Chia University, Taichung 40724, Taiwan; zipper0501@gmail.com

**Keywords:** blends, biodegradable polymer, crystallization behaviors, specific interactions, poly(3-hydroxybutyrate)

## Abstract

The green blends of an ionic liquid, 1-ethyl-3-propylimidazolium bis(trifluoromethanesulfonyl)imide {[EPrI][TFSI]}, and a biodegradable polymer, poly(3-hydroxybutyrate) (PHB), were investigated in this study. The influence of an ionic liquid on the crystallization behaviors of a biodegradable polymer was explored. In the blends, the presence of [EPrI][TFSI] decreased the *T*_g_ and *T*_m_ of PHB. Incorporating [EPrI][TFSI] in the blends reduced the degree of crystallinity of PHB, inferring that the [EPrI][TFSI] weakened the crystallization of PHB. It further showed retarded isothermal and non-isothermal crystallization for PHB with the presence of [EPrI][TFSI]. The smaller K and 1/*t*_0.5_ estimated by the Avrami equation for the blends indicated that [EPrI][TFSI] weakened the isothermal crystallization of PHB with exhibiting the slower crystallization rate. The present study also discussed non-isothermal crystallization of the blends. We found that the Mo model, which is generally used to discuss the non-isothermal crystallization, adequately described the non-isothermal behaviors of the [EPrI][TFSI]/PHB blends. By increasing the [EPrI][TFSI] content, the rate-related parameter *F*(*T*) systematically increased, inferring a decreased crystallization rate of PHB with the addition of [EPrI][TFSI] in the blends. The FTIR results suggested an ion–dipole interaction between [EPrI][TFSI] and PHB. This proposes the occurrence of possible complexation between [EPrI][TFSI] and PHB.

## 1. Introduction

Conventional petrochemical-based plastics have caused the environmental pollution for our Earth in the last several decades because of their lower degree of biodegradation [1,2]. Recently, much attention has been paid to the biodegradable polymers with the rise of concern about the environmental protection [3,4,5,6,7]. The biodegradable polymers can be mainly classified into the biosynthetic biodegradable polymers and chemosynthetic biodegradable polymers [8,9]. Among them, bacterially-synthesized poly(3-hydroxybutyrate) (PHB) is probably one of the most extensively studied biodegradable polymers. PHB has attracted much interest because of its biocompatibility and biodegradability. Therefore, PHB can be applied for both ecological and biomedical end-uses [10].

In general, PHB presents higher brittleness and a narrow processing window. The brittleness of PHB is mainly associated with its degree of crystallization, and the narrow processing window is due to the poor thermal stability between the processing temperature and melting temperature of PHB. The brittleness and narrow processing window of PHB have been the problems to extend the application of PHB in various fields [11,12]. Blends and copolymerization are often adopted to overcome the shortcomings of PHB [13,14]. It has been found that PHB is miscible with the small molecular additive, diglycidyl ether of bisphenol A (DGEBA) [15]. For the polymer blends of PHB, PHB are miscible with poly(*p*-vinylphenol) (PVPh) [16], poly(vinyl acetate) (PVAc) [17], starch acetate (SA) [18], and so forth. Silva et al. [15] have studied the miscibility in the PHB/DGEBA system. They found that the *T*_g_ of PHB decreased with increasing the DGEBA content. Zhang and coworkers [18] have reported the non-isothermal crystallization behaviors of the PHB/SA blends. It was revealed that the crystallization of PHB was hindered by the addition of SA. Applications and properties of the PHB (PHA) blends have also been widely discussed [19,20]. On the other hand, the poly(hydroxybutyrate-*co*-hydroxyvalerate) [P(HB-*co*-HV)] [21] and poly(hydroxybutyrate-*co*-hydroxyhexanoate) [P(HB-*co*-HH)] [22] are the two of common copolymers of PHA family. They were synthesized to improve the physical properties of PHB. Gunaratne et al. [21] have investigated the isothermal crystallization behaviors of the PHB copolymer, P(HB-*co*-HV), with various HV content. The results showed that the increased HV content decreased the isothermal crystallization rate of PHB.

The biodegradability and biorenewability of the green materials are important issues because of the considerations on environmental protection, ecotoxicity, and biotoxicity [23,24]. Ionic liquids (ILs), which are recognized as the environment-friendly green materials, have attracted intensive interest in several fields of chemical industry. It presents remarkable properties such as non-flammability, negligible vapor pressure, high ionic conductivity, thermal stability, and wide electrochemical window, etc. For the structural feature of the ILs, ILs are composed of dissociated organic cations and organic/inorganic anions. Furthermore, it could easily modify ILs through changing the structure of cations or anions, which will broaden their application fields [25]. Relevant discussions on the ILs are still necessary to explore the novel application of the ILs.

Crystallization behaviors are always important and critical for the studies of the biodegradable polymers. Crystallization behaviors could strongly associate with the physical properties and biodegradability of biodegradable polymers [26]. However, to the best of our knowledge, no report has mainly attempted to explore the effect of ILs on the crystallization behaviors of the biodegradable polymers. This work investigated the blends composed of an ionic liquid and a biodegradable polymer. The aim of this work is to discuss the influence of IL on the crystallization behaviors and kinetics of the biodegradable polymer. We studied the crystallization behaviors and kinetics of the blends comprising an ionic liquid, 1-ethyl-3-propylimidazolium bis(trifluoromethanesulfonyl)imide {[EPrI][TFSI]}, and PHB. The choice of [EPrI][TFSI] was considered by the following reasons: (i) [EPrI][TFSI] is a novel functional ionic liquid [27]. It is a potential green additive of polymer in the future, and relevant studies are interesting in the scientific point of view; (ii) it has been shown that the interactions existed between the ionic liquid with imidazolium cation ring and the polymer containing polar function group [28,29]. In our study, the [EPrI][TFSI] contains imidazolium cation ring and the PHB contains polar carbonyl group. Therefore, it may form interactions between [EPrI][TFSI] and PHB, and [EPrI][TFSI] may cause obvious changes in the crystallization behaviors of PHB; (iii) most ionic liquids are hydrophilic, thus the novel hydrophobic ionic liquids are particular attractive to scientists in different research fields. [EPrI][TFSI] is a novel hydrophobic ionic liquid [27]. The blends containing [EPrI][TFSI] can be less moisture sensitive. We found that [EPrI][TFSI] decreased the *T*_g_ and crystallinity of PHB. The crystallinity of PHB was reduced from 24.63% to 13.86% with adding 40 wt % [EPrI][TFSI] in the blends. Incorporating the ionic liquid [EPrI][TFSI] in the blends would reduce the crystallinity of PHB. It could further lower the brittleness of PHB to broaden its future applications. The influence of [EPrI][TFSI] on the crystallization behaviors of PHB was thoroughly discussed by the studies of isothermal and non-isothermal crystallization kinetics. The presence of [EPrI][TFSI] in the blends would reduce the crystallization of PHB.

## 2. Materials and Methods 

### 2.1. Materials and Blend Preparation

Poly(3-hydroxybutyrate) (PHB) with *M*_w_ = 500,000 g/mol was obtained from Polysciences Inc. (Warrington, PA, USA). An ionic liquid, 1-ethyl-3-propylimidazolium bis(trifluoromethanesulfonyl)imide, [EPrI][TFSI], was provided by Department of Chemical and Materials Engineering, National Yulin University of Science and Technology, Yunlin, Taiwan, Prof. Tzi-Yi Wu’s laboratory in collaboration with us. [EPrI][TFSI] was synthesized from 1-ethyl-3-propylimidazolium bromide [27]. The detailed preparation procedure of [EPrI][TFSI] is shown in the literature [27]. The [EPrI][TFSI]/PHB blending samples were prepared by the solution-casting method using chloroform as solvent. The film-casting procedure was performed by evaporating the solvent at 45 °C, followed by vacuum drying at 60 °C for at least 24 h. After evaporating the solvent completely, blending films showing different compositions were obtained.

### 2.2. Instruments and Experiments

Differential scanning calorimetry (DSC) (Perkin-Elmer DSC-8500, Perkin Elmer, Waltham, MA, USA) was utilized to study the thermal and crystallization behaviors of the blends. For *T*_g_ measurement, the blended samples were heated at a rate of 20 °C/min, and *T*_g_ values were noted as the onset of transition. Samples for studying the isothermal crystallization were first heated above the melting temperature of the PHB (~190 °C) and then rapidly cooled to various crystallization temperatures (*T*_c_) to crystallize. Exothermal curves for heat flow as a function of time were recorded to analyze the isothermal crystallization. On the other hand, for observing non-isothermal crystallization, all specimens were first annealed at 190 °C for 3 min and then cooled at different cooling rates to enable investigating non-isothermal crystallization.

Scanning electron microscopy (SEM) (Hitachi S3000, Hitachi, Tokyo, Japan) was carried out to confirm the phase morphology with greater magnification. Blended films for SEM observation were solution-casted to be thick enough so that the fracture surface of the thickness (cross-section) could be conveniently examined. Before SEM observation, the fractured blend samples were coated with gold by vapor deposition using vacuum sputtering.

Fourier-transform infrared spectrometer (FTIR) (Perkin-Elmer Frontier^TM^, Perkin Elmer, Waltham, MA, USA) was used to explore the interactions among the constituents. It recorded all spectra at a resolution of 4 cm^−1^ and an accumulation of 64 scans in the range of 400~4000 cm^−1^. The films for measurements were prepared by dropping the blended solution onto KBr pellets, followed by vacuum drying at 60 °C for at least 24 h to remove residual solvent. The film thickness was controlled so that the IR measurements could be properly processed. 

## 3. Results and Discussion

### 3.1. Phase Morphology and Thermal Properties of [EPrI][TFSI]/PHB Blends

**Figure 1:**
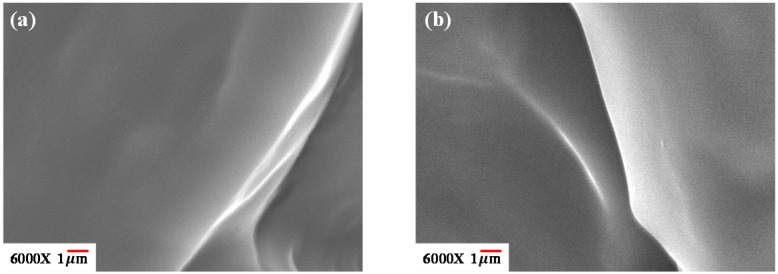
SEM cross-section morphologies for [EPrI][TFSI]/PHB blends with the compositions (**a**) 20/80 and (**b**) 40/60.

The preliminary results of optical microscopy (OM) showed that [EPrI][TFSI] could be well-mixed with PHB when the maximum content of [EPrI][TFSI] was 40 wt % in the blends (in brief, not shown here). Therefore, this study mainly discussed the [EPrI][TFSI]/PHB blends when the [EPrI][TFSI] content was limited, with the maximum content of 40 wt % in the blends. Since scanning electron microscopy (SEM) can provide a high-resolution image to confirm phase morphology, we utilized it to study the [EPrI][TFSI]/PHB blends. The fracture surfaces of blending samples were observed and the phase morphology of the [EPrI][TFSI]/PHB blends was resolved. Typical SEM results for the [EPrI][TFSI]/PHB blends are discussed herein. Figure 1 presents SEM images for [EPrI][TFSI]/PHB blends with the compositions: (a) 20/80 and (b) 40/60. As shown in Figure 1, SEM reveals a homogeneous morphology rather than the morphology with phase separation for the blends. 

**Figure 2:**
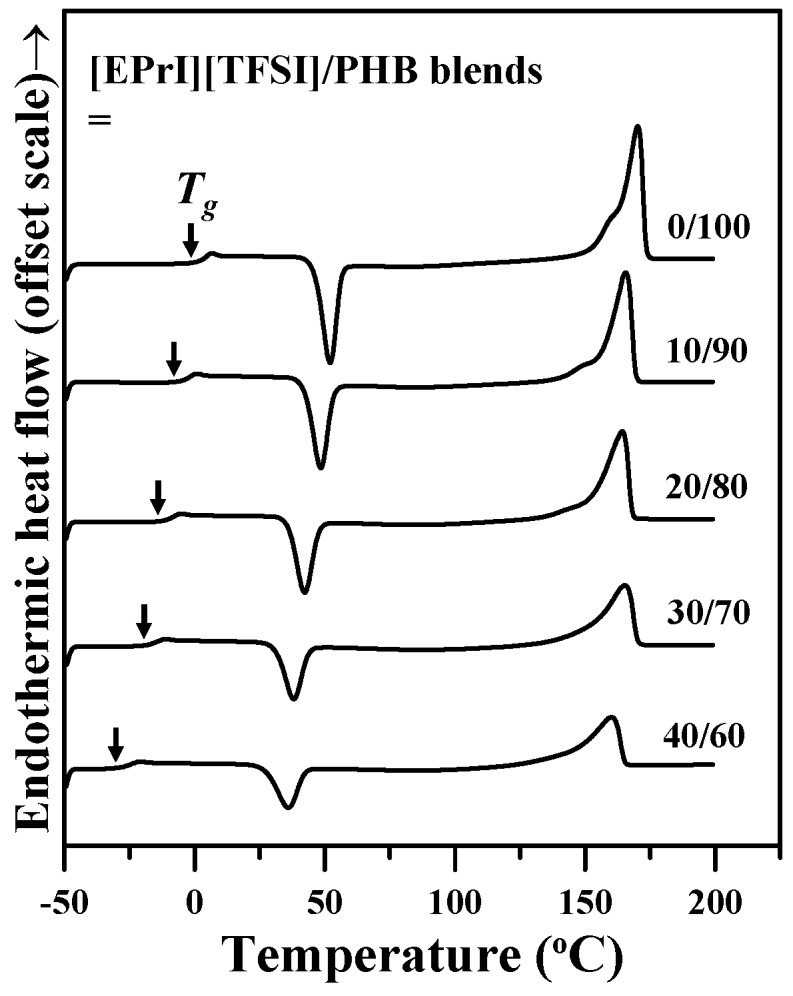
DSC thermograms of [EPrI][TFSI]/PHB blends with various compositions (wt %).

The thermal behaviors of [EPrI][TFSI]/PHB blends were also investigated by DSC. Blending samples were first melted at the temperature just above PHB’s *T*_m_ and then quenched for DSC measurements. Figure 2 shows the sequential DSC scans after melting/quenching each blending sample. As shown in Figure 2, all thermograms exhibited only one single *T*_g_ (arrow-marked) for each of the [EPrI][TFSI]/PHB blends with various compositions.

In addition, the *T*_g_s and *T*_m_s of [EPrI][TFSI]/PHB blends are relatively lower than that of neat PHB, as shown in Figure 2. DSC results demonstrated that the [EPrI][TFSI] in the blends influenced the thermal properties of PHB. It might further suggest that [EPrI][TFSI] moderately weakened the intramolecular interactions among PHB chains and decreased the chain cohesion of PHB as a molecular diluent. This phenomenon would influence the physical properties of PHB in the blends. Similar situations have been found in the literature reporting for other polymer-diluent pairs [30]. The peaks in the range of 25 to 50 °C in Figure 2 are the cold crystallization peaks of PHB in the blends. The degree of crystallinity (*X_c_*) was also evaluated by the melting enthalpy (Δ*H_m_*) measured by DSC. For each of the compositions in the blends, the value of Δ*H_m_* was calculated by the area of its melting transition shown in Figure 2. It should note that the Δ*H_m_* values were normalized by the content of PHB in the blends. The *X_c_* of neat PHB and the [EPrI][TFSI]/PHB blends were further estimated by the following equation:

(1) $$X_{C}=\;\frac{\Delta H_{m}}{\Delta H_{m}^{0}}\times 100\%$$

where $\Delta H_{m}^{0}$ is the melting enthalpy for 100% crystalline PHB. The $\Delta H_{m}^{0}$ value of neat PHB is 146.51 J/g [31]. Table 1 tabulates the *X_c_* and $\Delta H_{m}^{0}$ values of the [EPrI][TFSI]/PHB blends. From the results, the *X_c_* value of PHB decreased as the [EPrI][TFSI] increased. The *X_c_* value fell by 24.63% to 13.86% with the addition of [EPrI][TFSI] in the blends. This infers that the addition of [EPrI][TFSI] in PHB would reduce and weaken the crystallization of PHB. To further explore the influence of [EPrI][TFSI] on the crystallization behaviors of PHB, discussions on the isothermal crystallization and non-isothermal crystallization will be performed in following sections.

### 3.2. Study of Isothermal and Non-Isothermal Crystallization Kinetics in [EPrI][TFSI]/PHB Blends

#### 3.2.1. Isothermal Crystallization Kinetics

**Figure 3:**
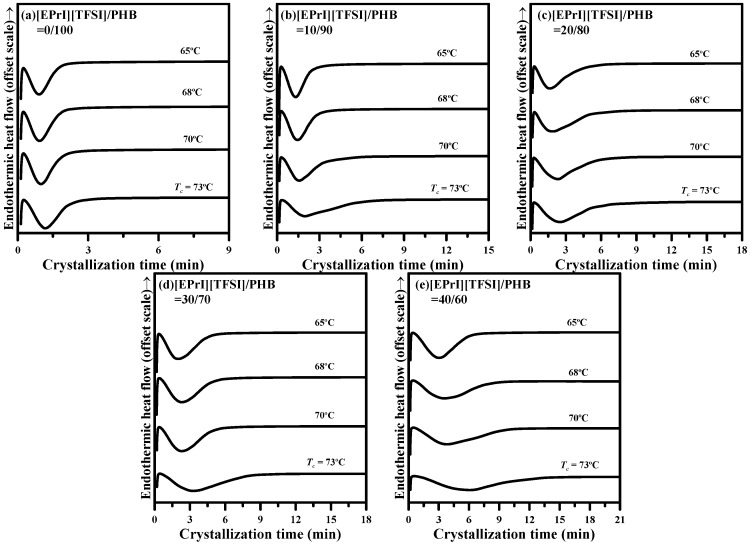
DSC isothermal crystallization thermograms of [EPrI][TFSI]/PHB blends with different compositions: (**a**) 0/100; (**b**) 10/90; (**c**) 20/80; (**d**) 30/70; and (**e**) 40/60.

Figure 3 shows the DSC traces for neat PHB and the [EPrI][TFSI]/PHB blends. Blending samples were heated above the melting temperature of the PHB (~190 °C) and held for 3 min to erase any thermal history. Subsequently, the samples were rapidly cooled to various crystallization temperatures (*T*_c_ = 65, 68, 70, 73 °C) and then maintained at *T*_c_ to crystallize isothermally.

The well-known Avrami equation [32,33] was used to analyze the isothermal crystallization kinetics of the [EPrI][TFSI]/PHB blends. The Avrami equation is given as follows:

(2) $$\ln\left(1-X_{t}\right)=-kt^{n}$$

where *X_t_* is the relative degree of crystallinity at time *t*, the exponent *n* is a constant with a value depending on the mechanism of crystallization, and the parameter *k* is a rate constant. In general, the larger *k* value implies the crystallization process with a faster crystallization rate, and the smaller *k* value means the crystallization showing a slower crystallization rate. The log-log representation of the Avrami equation is also presented as the following equation:

(3) $$\log\left[-\ln\left(1-X_{t}\right)\right]=\log k+n\log t$$

**Figure 4:**
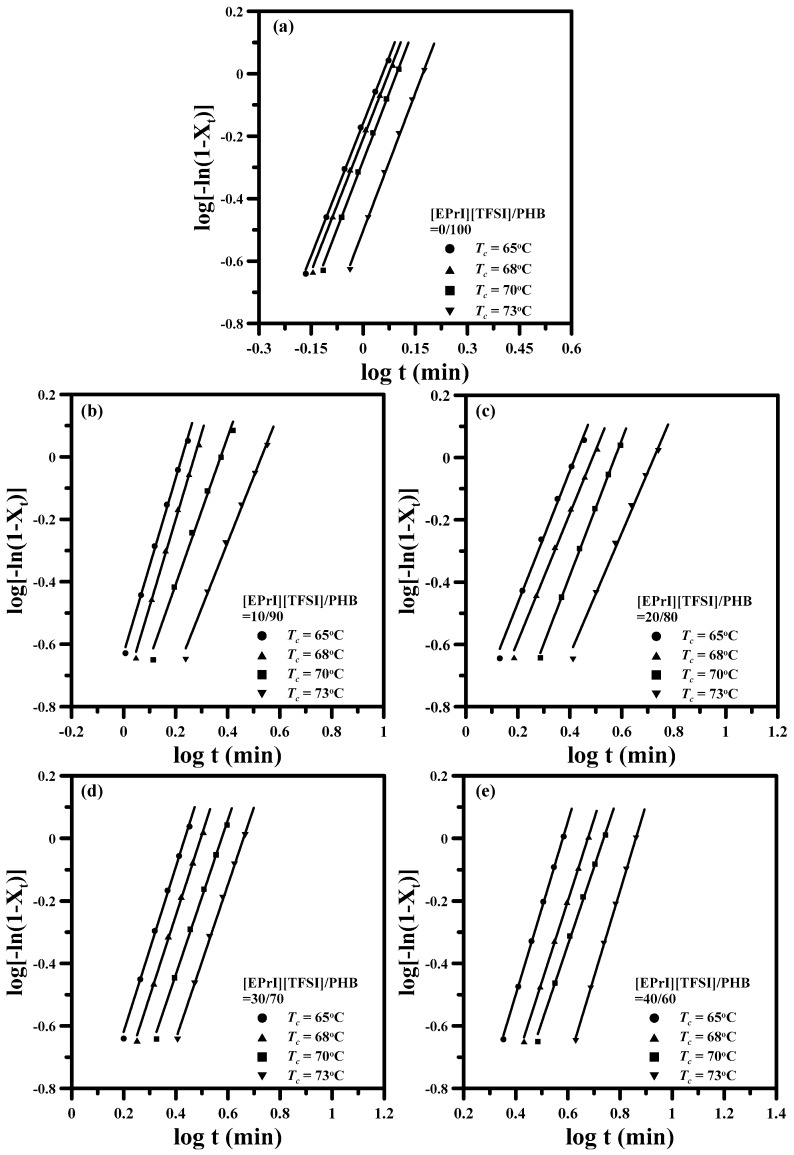
Avrami plots for isothermal crystallization of [EPrI][TFSI]/PHB blends with different compositions: (**a**) 0/100; (**b**) 10/90; (**c**) 20/80; (**d**) 30/70; and (**e**) 40/60 (crystallization temperatures are indicated in each graph).

Figure 4 shows the log-log plots of Avrami model for neat PHB and the [EPrI][TFSI]/PHB blends at different *T*_c_s. It displays that the isothermal crystallization behaviors of neat PHB and [EPrI][TFSI]/PHB blends can be described well by the Avrami equation. According to the fitting results, it could estimate the values of Avrami exponent (*n*) and crystallization rate constant (*k*). 

Table 2 shows relative parameters estimated by the fitting results. Meanwhile, the crystallization half-time (*t*_0.5_), which is defined as the time at which the extent of crystallization is 50%, can also be determined from the estimated kinetic parameters according to the following form:

(4) $$t_{0.5}={\left(\frac{ln2}{k}\right)}^{1/n}$$

Table 2 also lists the values of *t*_0.5_ for neat PHB and the [EPrI][TFSI]/PHB blends. In general, the larger 1/*t*_0.5_ value infers the crystallization with a faster crystallization rate, and the smaller 1/*t*_0.5_ value means the crystallization showing a slower crystallization rate. The values of n for all the blends containing different amounts of [EPrI][TFSI] lie between 2 and 3, indicating that the addition of [EPrI][TFSI] would not significantly affect the crystallization mechanism of PHB [34,35]. However, the values of k and 1/*t*_0.5_ decreased as the [EPrI][TFSI] content increased. This situation implies that the presence of [EPrI][TFSI] in the blends weakened the isothermal crystallization kinetic and decreased the crystallization rate of PHB. That is, the isothermal crystallization of PHB would be reduced, exhibiting a slower crystallization rate by the addition of [EPrI][TFSI] in the blends. 

#### 3.2.2. Non-Isothermal Crystallization Kinetics

**Figure 5:**
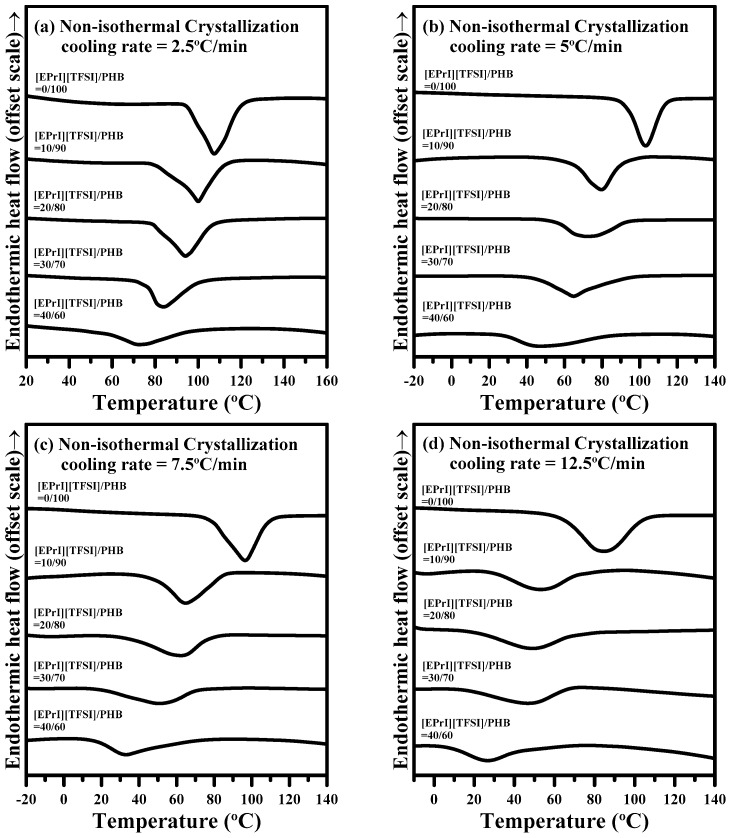
DSC results of non-isothermal crystallization of [EPrI][TFSI]/PHB blends with a different cooling rate: (**a**) 2.5 °C/min; (**b**) 5 °C/min; (**c**) 7.5 °C/min; and (**d**) 12.5 °C/min.

**Figure 6:**
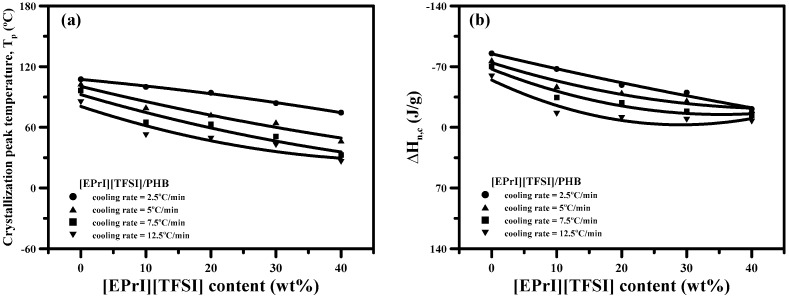
(**a**) Plot of *T*_p_-v.s.-[EPrI][TFSI] content in the blends with a different cooling rate; and (**b**) plot of Δ*H*_n,c_-v.s.-[EPrI][TFSI] content in the blends with a different cooling rate.

**Figure 7:**
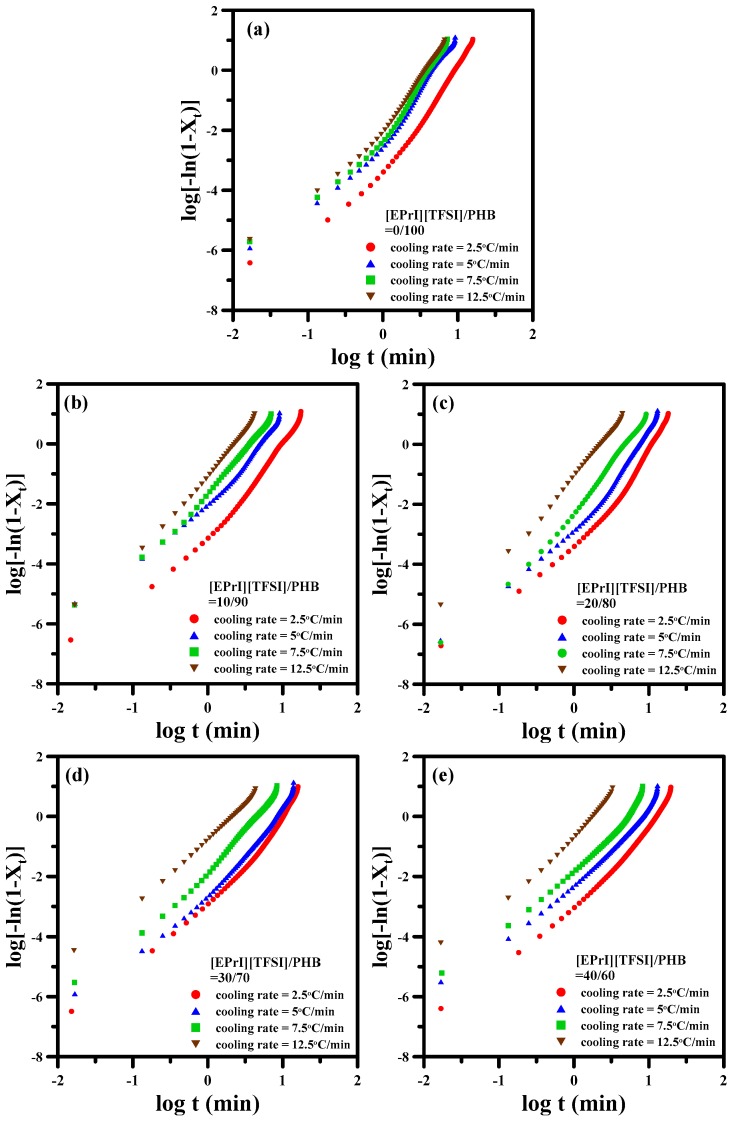
Avrami plots of log[−ln(1 − *X_t_*)]-v.s.-log(*t*) for the non-isothermal crystallization of [EPrI][TFSI]/PHB blends with different compositions: (**a**) 0/100; (**b**) 10/90; (**c**) 20/80; (**d**) 30/70; and (**e**) 40/60.

In this study, we also investigated the non-isothermal crystallization kinetics of [EPrI][TFSI]/PHB blends to further explore the influence of [EPrI][TFSI] on the crystallization behaviors of PHB. Figure 5 shows the DSC thermograms for neat PHB and the [EPrI][TFSI]/PHB blends at different cooling rates of 2.5, 5, 7.5 and 12.5 °C/min. The peak temperature of non-isothermal crystallization (*T*_p_) and the heat of non-isothermal crystallization (Δ*H*_n,c_) were analyzed. We summarized the main results of Figure 5 in Figure 6. Figure 6a shows that the *T*_p_ shifted to a lower temperature as the [EPrI][TFSI] content was increased, regardless of changes in the cooling rate. Figure 6b shows a similar tendency for the values of Δ*H*_n,c_ (heat of non-isothermal crystallization), which decreased as the [EPrI][TFSI] content was increased. These two phenomena preliminary showed that [EPrI][TFSI] might retard and decline the non-isothermal crystallization of crystalline PHB. The non-isothermal crystallization kinetics could be described by different models including the modified Avrami equation [36], Ozawa analysis [37], and Mo method [38]. The Avrami equation modified by Jexiorny [36] was firstly used to analyze the non-isothermal crystallization kinetics of the [EPrI][TFSI]/PHB blends. The modified Avrami equation suggests that the non-isothermal crystallization with a fixed cooling rate comprises a series of infinite small isothermal crystallization steps. The kinetics of the overall non-isothermal crystallization rate is expressed as the following equation:

(5) $$X_{t}=1-exp\left(-Z_{t}t^{n^{\prime }}\right)$$

where the exponent *n*′ is a constant depending on the type of nucleation and crystal growth dimension. Also, *Z_t_* is the crystallization rate constant related to the nucleation and growth parameters. In non-isothermal crystallization, time (*t*) can be related to temperature (*T*) as the following relationship:

(6) $$t=\frac{T_{0}-T}{\Phi }$$

where Φ is the cooling rate. It can obtain the values of *n*′ and *Z_t_* from the slope and intercept of the straight regime from plots of log[−ln(1 − *X_t_*)]-v.s.-log(t). Figure 7 illustrates the plots of log[−ln(1 − *X_t_*)]-v.s.-log(*t*) for different [EPrI][TFSI]/PHB blends with changing the cooling rate. Each curve shows non-linear relationship, inferring that the modified Avrami equation was not suitable for describing the non-isothermal crystallization kinetics of [EPrI][TFSI]/PHB blends. 

The Ozawa equation was also used to discuss the non-isothermal crystallization kinetics in the blends. Ozawa [37] has extended the Avrami equation to further investigate the non-isothermal crystallization process. It was considered that the non-isothermal crystallization process should consist of a large number of infinitesimal isothermal crystallization steps. The Ozawa equation for studying the non-isothermal crystallization process is expressed as the following equation:

(7) $$X_{T}=1-\exp\left(-\frac{K\left(T\right)}{\Phi^{m}}\right)$$

where *X_T_* is the relative crystallinity at a temperature *T*, *K*(*T*) is the cooling function for the overall crystallization rate, and Φ is the cooling rate and *m* is the Ozawa exponent. The Ozawa exponent depends on the dimension of the crystal growth. One could rearrange the Ozawa equation by taking logarithms on both sides to show the following form:

(8) $$\log\left[-\ln\left(1-X_{T}\right)\right]=\log K\left(T\right)-m\log\Phi$$

**Figure 8:**
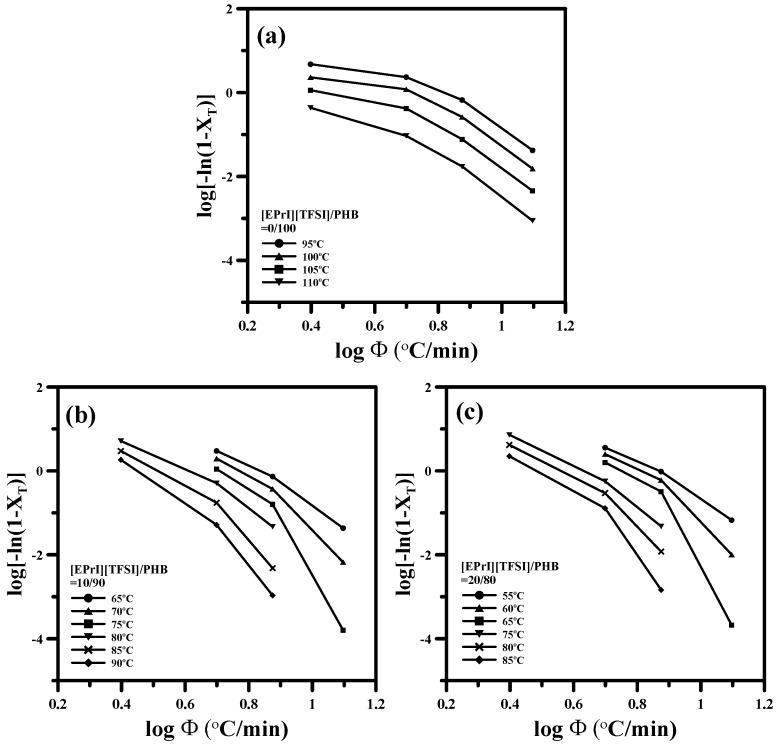

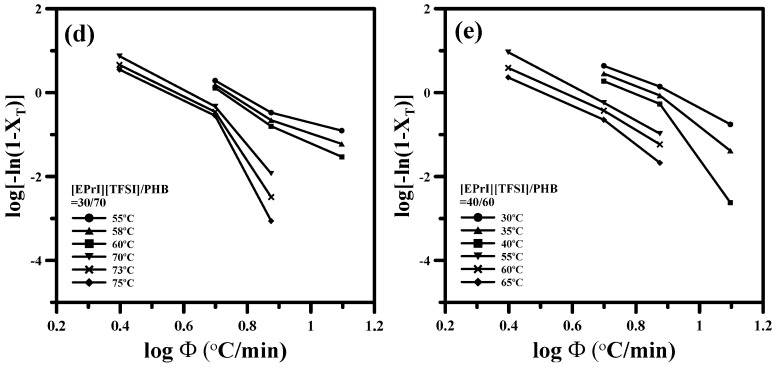
Ozawa plots of log[−ln(1 − *X_T_*)]-v.s.-logΦ for the non-isothermal crystallization of [EPrI][TFSI]/PHB blends with different compositions: (**a**) 0/100; (**b**) 10/90; (**c**) 20/80; (**d**) 30/70; and (**e**) 40/60.

Figure 8 shows the plots of log[−ln(1 − *X_T_*)]-v.s.-logΦ for different [EPrI][TFSI]/PHB blends. It displays that all plots in Figure 8 are not linear. This result suggests that the Ozawa equation fails to provide a suitable description for the non-isothermal crystallization of [EPrI][TFSI]/PHB blends. The above results demonstrate that the Avrami model and Ozawa model cannot adequately describe the non-isothermal crystallization of [EPrI][TFSI]/PHB blends. The failure of applying the Avrami model and Ozawa model in describing the non-isothermal crystallization behaviors might be attributed to the possible secondary crystallization in the blends. A similar situation has been also reported in the literature [39,40].

**Figure 9:**
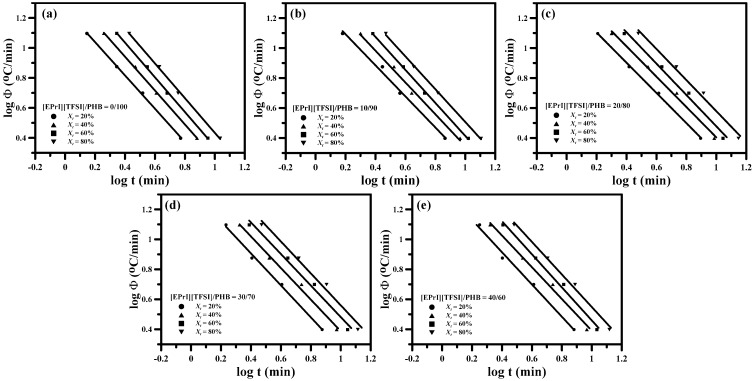
Mo model plots of logΦ-v.s.-log(*t*) for non-isothermal crystallization of [EPrI][TFSI]/PHB blends with different composition: (**a**) 0/100; (**b**) 10/90; (**c**) 20/80; (**d**) 30/70; and (**e**) 40/60.

Mo and coworkers [38] have also derived a model to study the non-isothermal crystallization. We further applied the Mo model to investigate the non-isothermal crystallization kinetics of the blends comprising [EPrI][TFSI] and PHB. The equation is given below:

(9) logΦ=−alogt+logF(T)

where logF(*T*) = [*K*(*T*)/*k*]^1/*m*^ and the *M*o index “a” is the ratio between the Avrami exponent (*n*) and Ozawa exponent (*m*). The F(*T*) refers to the value of the cooling rate required to reach a defined degree of crystallinity at a certain temperature in the unit crystallization time. It has been shown that the F(*T*) could be related to the rate of non-isothermal crystallization [41], and a higher value of F(*T*) could be associated with a retarded crystallization with lower crystallization rate. The parameters a and F(*T*) can be determined by the slope and intercept of the plot of logΦ versus log(*t*) at defined relative crystallinity. The plot between logΦ and log(*t*) gives a straight-line relationship for the blends of [EPrI][TFSI]/PHB as shown in Figure 9, suggesting that the Mo model can properly describe the non-isothermal crystallization kinetics of [EPrI][TFSI]/PHB blends. 

We further tabulated the parameters estimated by the Mo model in Table 3. At the same extent of crystallinity, one can find that the F(*T*) value increases systematically with increasing the amount of [EPrI][TFSI] in the blends. The relevant analyses demonstrated that the presence of [EPrI][TFSI] in the blends decreased the non-isothermal crystallization rate of PHB. The [EPrI][TFSI] in the blends would cause a reduction in proceeding non-isothermal crystallization of PHB. We suggest that the efficient dilution effect exerted by the [EPrI][TFSI] in the blends retarded the non-isothermal crystallization of PHB. That is, [EPrI][TFSI] diluted the polymer chains of PHB and significant enlarged the inter-chain distance of PHB by the efficient dilution. The corporative diffusion and aggregation of PHB for proceeding crystallization were diminished, and the crystallization of PHB was then retarded and declined. By the above results, it suggests that the [EPrI][TFSI] significantly influenced the crystallization behaviors of PHB in the blends, and it retarded the isothermal and non-isothermal crystallization of PHB by the dilution effect.

### 3.3. FTIR Characterization on [EPrI][TFSI]/PHB Blends

**Figure 10:**
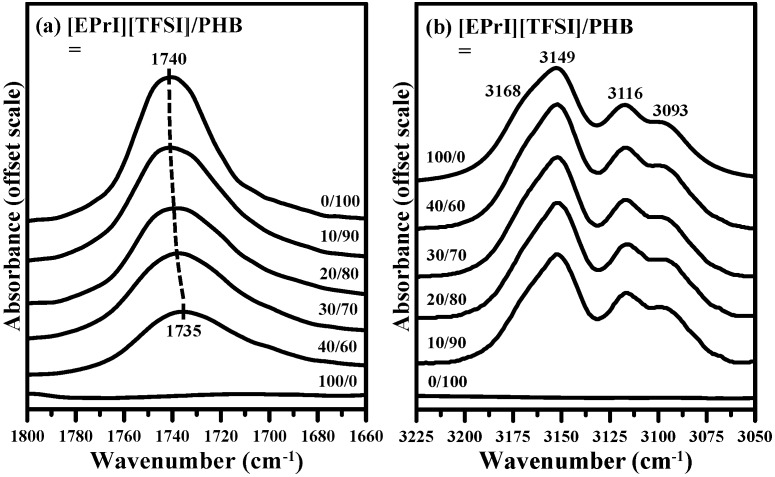
(**a**) Carbonyl stretching region in FTIR spectra of [EPrI][TFSI]/PHB blends; and (**b**) FTIR spectra in the C–H stretching vibrational region of imidazolium cation ring for [EPrI][TFSI]/PHB blends with different compositions.

The FTIR spectra of neat PHB, neat [EPrI][TFSI] and [EPrI][TFSI]/PHB blends are given in Figure 10. Figure 10a shows the IR spectra of carbonyl stretching region (1800–1660 cm^−1^) for [EPrI][TFSI]/PHB blends. The absorption peak of neat PHB was found at 1740 cm^−1^. As the [EPrI][TFSI] content was increased, the peak shifted to 1735 cm^−1^, suggesting the presence of interactions between PHB and [EPrI][TFSI]. Samples were kept at the molten amorphous state for measurements to avoid the complexity of crystallization. Figure 10b shows the IR C–H stretching vibration band (3225–3050 cm^−1^) of the imidazolium cation ring in [EPrI][TFSI]. The C–H stretching vibration band from 3225 to 3050 cm^−1^ for the imidazolium cation ring was assigned in the literature [42]. For the neat [EPrI][TFSI], a shoulder at 3168 cm^−1^ and three peaks at 3149, 3116, and 3093 cm^−1^ can be shown in Figure 10b. A detail discussion of the IR results of imidazolium cation ring was performed by spectral deconvolution. 

**Figure 11:**
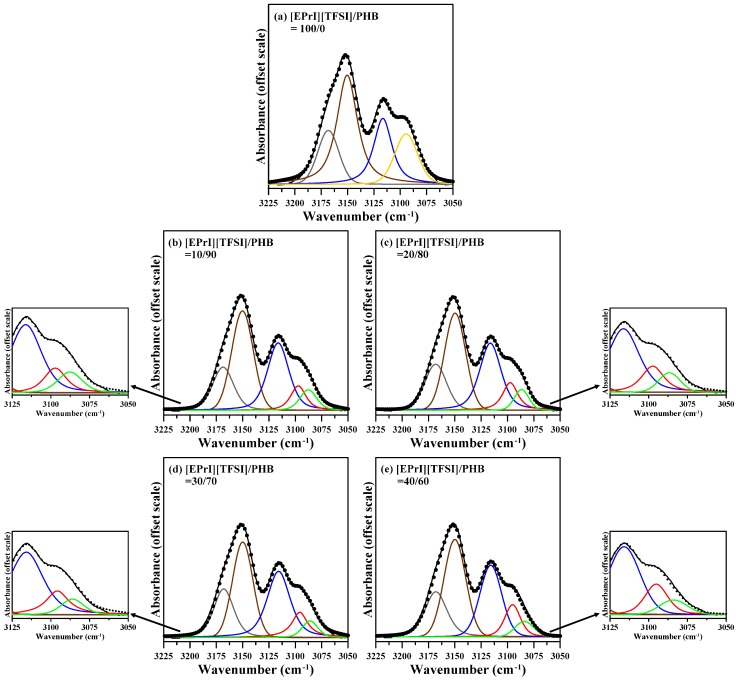
Deconvoluted FTIR spectra in the C–H stretching vibrational region of imidazolium cation ring for [EPrI][TFSI]/PHB blends with different compositions: (**a**) 100/0; (**b**) 10/90; (**c**) 20/80; (**d**) 30/70; and (**e**) 40/60.

Figure 11 shows deconvoluted IR spectra for the C–H stretching vibrational band of imidazolium cation ring with different compositions in the blends. It displays that for the [EPrI][TFSI]/PHB blends the peak at 3093 cm^−1^ split into two peaks as at the 3087 and 3097 cm^−1^. The smaller inserted diagrams are the plots with expanded *x*-axis (from 3125–3050 cm^−1^) for relevant compositions. A similar situation has also been found in the blends of poly(vinylidene fluoride-*co*-hexafluoropropylene) (PVDF-HFP) and an ionic liquid containing an imidazolium ring cation [42]. According to the literature [42], this feature of IR spectra could result from two forms of the conformation: (i) IL cation complexed with the polymer chain; and (ii) uncomplexed IL cation, and the absorption band at lower wavenumber (3087 cm^−1^) and higher wavenumber (3097 cm^−1^) could be assigned to the complexed and uncomplexed forms of the conformation, respectively. 

**Figure 12:**
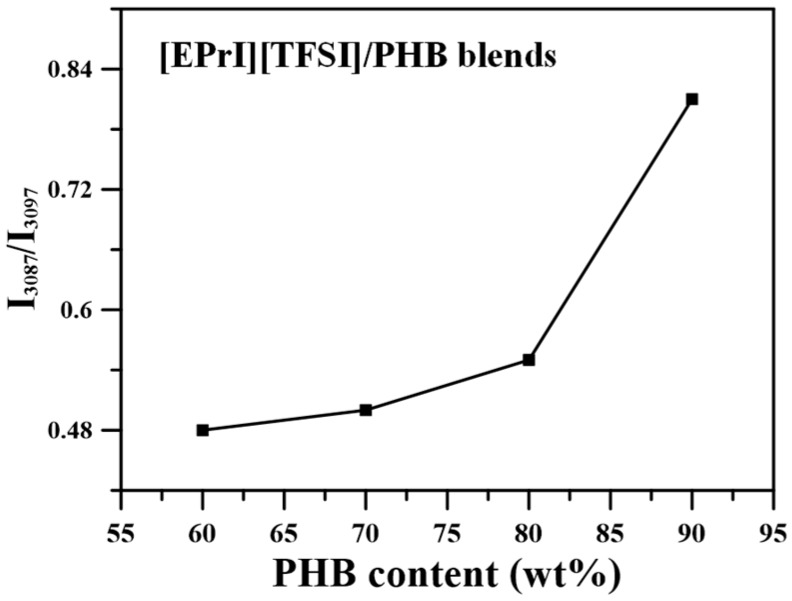
Plot of (I_3087_/I_3097_)-v.s.-PHB content for the [EPrI][TFSI]/PHB blends.

**Figure 13:**
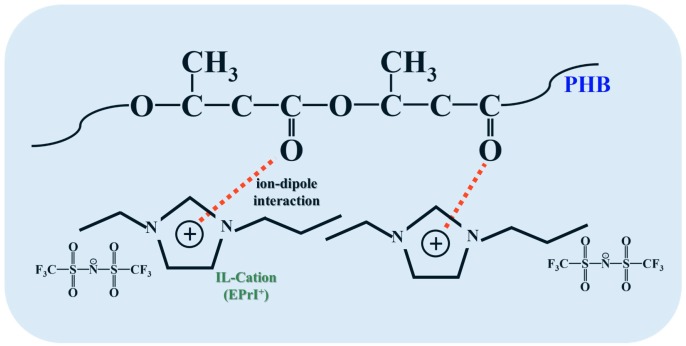
Illustration of the possible ion–dipole interaction between PHB and [EPrI][TFSI].

We further estimated relative ratios between the absorption intensity of 3087 cm^−1^ (*I*_3087_) to 3097 cm^−1^ (*I*_3097_). The values of (*I*_3087_/*I*_3097_) are presented in Figure 12. Figure 12 demonstrates the plot of (*I*_3087_/*I*_3097_)-v.s.-PHB content in the blends. The value of (*I*_3087_/*I*_3097_) increased when the PHB content was increased. In other words, the value of (*I*_3087_/*I*_3097_) increased when the [EPrI][TFSI] content was decreased. For the present blending system, one could suggest that the inter-association between PHB and [EPrI][TFSI] competed with the intra-association of the ILs in the blends. In the blends with higher PHB content (or lower [EPrI][TFSI] content), the inter-association between PHB and [EPrI][TFSI] would dominate the interaction state because of the rich amount of PHB in the blends. This state of interaction promoted the formation of complexation between PHB and [EPrI][TFSI]. The above results infer that the complexation caused by the inter-association between [EPrI][TFSI] and PHB could be formed. The formation of the ion–dipole interactions between the [EPrI] cation of [EPrI][TFSI] and the carbonyl group of PHB could be proposed. Similar situation has also been reported for the interactions between a cation and a carbonyl-containing polymer in their blends [43]. Kuo et al. [43] have studied the blends comprising lithium salt (LiClO_4_) and poly(vinylpyrrolidone) (PVP). They have proposed that the complexation between LiClO_4_ and PVP should be resulted from the ion–dipole interactions between the cation of Li^+^ and the carbonyl group of PVP. In addition, according to the FTIR results in our blending system, it also suggests that the excess loading of [EPrI][TFSI] would not benefit the complexation. The illustration presenting the complexation between [EPrI][TFSI] and PHB is shown in Figure 13.

## 4. Conclusions

In this study, we discussed blends comprising ionic liquid and biodegradable polymer, and investigated the influence of ionic liquid on the crystallization behaviors of the biodegradable polymer. The ionic liquid, [EPrI][TFSI], decreased the *T*_g_ and *T*_m_ of PHB as a diluent. Additionally, with the presence of [EPrI][TFSI], the degree of the crystallinity of PHB was also decreased, inferring the declined crystallization of PHB exerted by [EPrI][TFSI]. Investigations on the isothermal crystallization and non-isothermal crystallization of [EPrI][TFSI]/PHB blends were also performed to further understand the influence of [EPrI][TFSI] on the crystallization behaviors of PHB. The Avrami equation was used to discuss the isothermal crystallization kinetics of the [EPrI][TFSI]/PHB blends. It was found that the presence of [EPrI][TFSI] influenced the isothermal crystallization of PHB in the blends. The [EPrI][TFSI] obviously weakened the isothermal crystallization kinetics of PHB by presenting a slower crystallization rate for the blends. This phenomenon was proven by the smaller K and 1/*t*_0.5_ values obtained by the Avrami equation. Furthermore, we also found that [EPrI][TFSI] retarded and declined the non-isothermal crystallization kinetic of PHB. We found that the non-isothermal crystallization of the [EPrI][TFSI]/PHB blends was described properly by the Mo model. The F(T) value estimated by the Mo model increased systematically with increasing the amount of [EPrI][TFSI] in the blends. This result demonstrated that the [EPrI][TFSI] in the blends decreased the non-isothermal crystallization rate of PHB. The polymer chains of PHB could be diluted by the [EPrI][TFSI] to enlarge the inter-chain distance of PHB. The corporative diffusion and aggregation of PHB for proceeding crystallization were diminished, and the crystallization of PHB was retarded and declined. The FTIR results suggested the occurrence of the ion–dipole interaction between [EPrI][TFSI] and PHB.

## Figures and Tables

**Table 1:** Δ*H**_m_*** and *X_c_* values of the [EPrI][TFSI]/PHB blends.

[EPrI][TFSI]/PHB (wt %)	Δ*H*_m_ (J/g)	*X_c_* (%)
0/100	36.08	24.63
10/90	32.27	22.03
20/80	30.42	20.76
30/70	23.67	16.16
40/60	20.30	13.86

**Table 2:** Crystallization parameters evaluated by using the Avrami equation for the [EPrI][TFSI]/PHB blends.

[EPrI][TFSI]/PHB (wt %)	*T*_c_ (°C)	*n*	*k* (min^−n^)	*t*_0.5_ (min)	1/*t*_0.5_ (min^−1^)
0/100	65	2.84	0.695	0.99	1.01
	68	2.84	0.620	1.04	0.96
	70	2.90	0.527	1.09	0.91
	73	2.93	0.314	1.31	0.76
10/90	65	2.77	0.234	1.48	0.68
	68	2.75	0.175	1.65	0.61
	70	2.38	0.131	2.01	0.50
	73	2.12	0.076	2.84	0.35
20/80	65	2.13	0.129	2.20	0.45
	68	2.05	0.100	2.57	0.39
	70	2.23	0.072	2.76	0.36
	73	2.27	0.055	3.05	0.33
30/70	65	2.50	0.076	2.42	0.41
	68	2.58	0.053	2.71	0.37
	70	2.48	0.037	3.26	0.31
	73	2.47	0.023	3.97	0.25
40/60	65	2.77	0.025	3.32	0.30
	68	2.60	0.018	4.07	0.25
	70	2.48	0.015	4.69	0.21
	73	2.76	0.004	6.47	0.15

**Table 3:** Non-isothermal crystallization parameters estimated by the Mo model.

[EPrI][TFSI]/PHB (wt %)	*X_t_* (%)	a	*F*(*T*)
0/100	20	1.11	18.24
	40	1.13	24.77
	60	1.15	31.48
	80	1.15	39.08
10/90	20	1.04	19.91
	40	1.06	25.53
	60	1.10	32.73
	80	1.09	39.90
20/80	20	1.00	20.18
	40	1.01	26.06
	60	1.04	33.19
	80	1.04	41.30
30/70	20	1.06	21.53
	40	1.05	27.61
	60	1.05	34.20
	80	1.07	42.17
40/60	20	1.08	22.13
	40	1.05	28.12
	60	1.10	36.48
	80	1.09	43.15
